# Deep venous thrombosis after major abdominal surgery in a Ugandan hospital: a prospective study

**DOI:** 10.1186/1865-1380-6-43

**Published:** 2013-11-28

**Authors:** Andrew L Muleledhu, Moses Galukande, Patson Makobore, Tom Mwambu, Faith Ameda, Elsie Kiguli-Malwadde

**Affiliations:** 1 Department of Surgery, Mulago National Referral Hospital, P.O. Box 7072, Kampala, Uganda; 2 Department of Surgery, Makerere University College of Health Sciences, Mulago Hill Road, P.O. Box 7072, Kampala, Uganda; 3 Department of Radiology, Makerere University College of Health Sciences, P.O. Box 7072, Kampala, Uganda

**Keywords:** Deep venous thrombosis, Duplex Doppler ultrasound, Postoperative, Sub-Saharan Africa

## Abstract

**Background:**

Deep venous thrombosis (DVT) is a major cause of morbidity and mortality among postoperative patients. Its incidence has been reported to range between 16% and 38% among general surgery patients and may be as high as 60% among orthopaedic patients. The most important clinical outcome of DVT is pulmonary embolism, which causes about 10% of hospital deaths. In over 90% of patients, occurrence of DVT is silent and presents no symptoms until onset of pulmonary embolism and/or sudden death. The only effective way of guarding against this fatal condition is therefore prevention/prophylaxis. However, prophylaxis programs are usually based on the estimated prevalence of DVT in that particular community. There is currently no data concerning rates of postoperative DVT in Uganda.

The purpose of the study was therefore to determine the prevalence of DVT among postoperative patients at Mulago Uganda’s National Referral Hospital.

**Methods:**

A cross sectional descriptive study was conducted between March and June 2011. Eligible patients were identified and screened and patient details were collected. Clinical examinations were done on postoperative days (PODs) 1, 2, and 4 and Doppler ultrasounds were done on POD 7 and POD 21 to assess for DVT. Patients found with DVT were treated appropriately according to local treatment guidelines.

**Results:**

A total of 82 patients were recruited, 4/82 (5%) had DVT. The most common risk factor was cancer. The overall mean age was 45 years (range 20–83 years). The male to female ratio was 1.6:1. Participants with more than one risk factor for DVT were 16/82 (20%).

**Conclusions:**

Prevalence of DVT among major post-abdominal surgery patients was low (5%). Cancer was the most common associated factor apart from surgery.

## Background

Deep venous thrombosis (DVT) is a major cause of morbidity and mortality worldwide, with an annual incidence in the general population estimated to be approximately 1 in 1,000 in some populations; in the USA there are approximately 350,000 new cases of DVT annually [1]. Occurrence of DVT during hospital stay is even higher than in the general population; the incidence of this hospital-acquired DVT has been reported to account for about 50% of all cases of venous thromboembolism (VTE) [2]. This high incidence is due to the many risk factors that exist in patients admitted to hospitals. Among the most important risk factors are malignancies, vascular disease, trauma, and surgery, as well as other conditions that lead to prolonged hospitalisations.

Since surgery is an independent risk factor, postoperative patients are at a particularly high risk of developing DVT. Many of these patients have additional risk factors that may be the primary reason for the surgery; for example, cancer patients undergoing excision for a tumour or a patient with vascular disease undergoing vascular surgery. Patients admitted for surgery account for one quarter of all VTE cases [2].

Though most authors still regard venography as the gold standard for diagnosis, recent publications have found that Doppler ultrasound has very high sensitivity (up to 100%) and specificity (91.8%) [3]. Some authors have gone as far as referring to Doppler ultrasound as the current gold standard for diagnosis of DVT [4-6]. Many screening protocols currently combine the Wells clinical scoring system with D-dimer assays to exclude DVT [7].

In Uganda, like in many other countries in the East African region, DVT is not prioritized as a major problem among postoperative patients and the general sense is that the rate of postoperative DVT is not high. Prophylaxis against DVT is therefore not common practice. There is paucity of data about the prevalence of this potentially major cause of morbidity and mortality in Uganda [8].

This research set out to establish the prevalence of DVT among patients who underwent major abdominal surgery at a tertiary hospital in sub-Saharan Africa.

## Methods

### Study design and settings

This was a prospective study conducted in Mulago Hospital on the general surgical wards between March and June 2011. Mulago Hospital is the national referral hospital of Uganda and serves as the teaching hospital for the College of Health Sciences, Makerere University. The hospital is situated in Kampala City, the capital of Uganda, which serves a population of over 30 million people. It has a capacity of about 1,500 beds and admits about 40 surgical and trauma patients to its surgical emergency ward daily. There are five operating theatres for elective surgery and two theatres for emergency operations. Emergency theatres handle up to 10 to 15 operations per day including, but not limited to, general surgical, neuro, orthopaedic, ENT and urology cases.

### Recruitment of patients and data collection

All patients scheduled to undergo major abdominal surgery and who were 18 years old and above were recruited for the study. Patients were recruited from the emergency ward and general surgical wards. Patients were appropriately resuscitated and stabilized and relevant specialists were consulted depending on initial assessment. All postoperative patients who had had laparotomies were identified through patient case notes. Those patients who met the criteria were interviewed using a pretested questionnaire on the 1^st^ postoperative day (POD). Informed written consent was obtained from all patients. Patients on anticoagulant treatment, neurosurgical patients, and orthopaedic patients were excluded.

Patients were examined for signs of DVT or pulmonary embolism based on the Wells score [5]. The patients’ findings were then categorized as high (≥3) or non-high (≤2). Information regarding the duration of surgery and perioperative blood transfusion was obtained from the patients’ files and theatre records. Further clinical examination (based on the Wells score) of the patients was done on PODs 2 and 4. If at any time the patient scored high on the Wells score, he/she immediately underwent Doppler ultrasound, and if DVT was confirmed, appropriate treatment was instituted. Patients that did not develop clinical DVT while still admitted were scheduled to undergo duplex ultrasound on PODs 7 and 21.

All patients were assessed for DVT using standard Mulago Hospital protocol by a senior radiologist. All patients were scanned while lying flat on an examination bed, in supine position for the thigh veins and prone or decubitus position for the popliteal and calf veins. Unilateral and bilateral studies were done for patients with unilateral and bilateral symptoms, respectively. The venous system (including limbs, pelvis, and abdomen) was imaged systematically using a linear 7.5 to 10 MHz transducer of an ATL 1500 US machine (manufactured in 2000). Duplex scans were performed. The Doppler examination assessed the direction, velocity, and pattern of blood flow, and the venous and arterial vessels demonstrating the characteristic pattern. Normal venous vasculature shows venous flow at baseline and augmentation of flow with calf compression and phasic respiratory ventilation with increased flow during expiration. Augmentation helped to assess for obstruction distal to the probe. Respiratory variation helped to assess obstruction proximal to the probe. Hard copy sonograms for each scan were obtained for off-line interpretation and record keeping. The information was recorded on pre-coded data forms.

### Study variables

Study variables included age, sex, BMI, duration of immobilization (bed-bound duration), site of surgery, steroid use, length of operation, reference to start and stop time recorded in the theatre log and anaesthetic charts, and presence of DVT and DVT risk factors.

### Ethical considerations

Ethical approval was obtained from the institutional review board of the Makerere University College of Health sciences and the Mulago Hospital ethics committee. A written informed consent was obtained from the study participants.

## Results

During the 5-month study period, 92 patients who met the inclusion criteria were recruited into the study. Ten patients did not undergo the final Doppler ultrasound on POD 21 due to 3 deaths, 4 were lost to follow-up, and 3 were too sick to undergo further ultrasound examinations. The dead subjects were excluded from analysis because post-mortem examinations were not performed. In total, 82 patients were analysed for the study.

### Demographics

The male to female ratio was (M: F) 1.6:1. The age range was 20 to 83 years old with a mean of 45 years; 60% of the patients were above 40 years old; 74% (61/82) had a BMI (between 18.5 and 25) and two patients were obese (BMI >30).

### History

The most common risk factor was a history of cancer followed by use of steroids (Table 1); 80% (66/82) of patients had a positive history of factors associated with DVT.

**Table 1:** Characteristics of study participants in the Ugandan DVT study, 2012

**Characteristics**	**Number**
Age	45 years (20–83)
Duration of operation	Mean 82 minutes (45–170)
Duration of immobilization	≥3–64 days
	Mean 3.4 days
Risk factors for DVT	
Steroids use	12
Cancer diagnosis	26
Leg varices	2
Non insulin dependent diabetes mellitus	5
Smoking	10
HIV seropositive	10
BMI	
<18.5	2
18.5–25	30
25.1–30	31
>30	2
Number of risk factors per patient	
1	34
2	11
3	5

### Duration of operations and immobilisation

The mean duration of operation was 82 minutes (1 hour 22 minutes) with a range of 45 to 170 minutes. Overall, 78% of the patients were immobilised for 3 days or less. The longest period of immobilisation was 60 days in a patient who had exploratory laparotomy for an intra-abdominal lymphoma and developed an enterocutaneous fistula postoperatively. The average duration of immobilisation was 3.4 days.

### Prevalence of DVT

Four (5%) patients (three females and one male) had DVT. All had advanced malignancies, three of which originated in the pelvis. The patient with a non-pelvic tumour (advanced pancreatic carcinoma) was a 58-year-old female who also had diabetes mellitus, congestive heart failure, and was obese (BMI 39). All four patients were symptomatic; the onset of symptoms was within 2 days of surgery. The symptoms were limb swelling, oedema, and tenderness. The 80-year-old elderly female patient had a stage III tumour in the descending colon and was diagnosed with DVT on POD 7 using Doppler ultrasound. A 23-year-old man with advanced adenocarcinoma had a thrombus in the inferior vena cava extending into the common iliac veins bilaterally. In this patient, both common femoral veins and the distal vasculature was devoid of thrombus. The last patient was a 28-year-old woman with advanced rectal carcinoma and HIV whose surgery was performed for staging and a diverting colostomy. All patients had proximal DVT. The details of all patients that developed DVT are shown in Table 2.

**Table 2:** Summary of characteristics for participants who developed DVT, a Ugandan DVT study, 2012

**Characteristic**	**Patients**			
	**1**	**2**	**3**	**4**
Sex	F	F	F	M
Age	80	58	28	23
Cancer	Yes	Yes	Yes	Yes
Site of operation	Abdomen	Abdomen	Pelvic	Pelvic
Duration of operation (minutes)	70	55	60	150
Blood transfusion	Yes	Yes	No	Yes
BMI	19.2	38.9	24.7	22.5
Smoking	No	No	No	No
Steroid use	No	No	Yes	No
DVT history	No	No	No	No
HIV	No	No	Yes	No

## Discussion

We set out to establish the DVT rates among major abdominal general surgical patients at a tertiary hospital in Uganda. The prevalence of DVT was 5%, which is similar to that in studies performed in Nigeria [9] and among Hong Kong Chinese [10]. The mean age of 45 is considerably lower than in other studies in which the rates were high. Patients in the study by Prystowsky et al. [11] had a mean age of 41 and a DVT rate of 4%; patients in the study by Beyer et al. [12] had a mean age of 65 and an incidence of 24%. The mean age of the study participants relates to the mean age of the general population. In Germany, where Beyer conducted his study, 20% of the population is above the age of 65 compared to Uganda where only 3% of the population is above 65 years old [13]. The incremental risk associated with increasing age [14] indicates that the Ugandan population would have an increasing risk of DVT as the general population’s mean age increases. Indeed, the demographic composition in Uganda has been changing over the past few years with many people living longer. Life expectancy has improved from 45 years in 1995 to 53 years in 2009 [13].

Malignancy has been and continues to be strongly associated with increasing age worldwide. However, in Uganda, some cancers occur at ages much lower than those reported internationally. Colorectal and breast cancer have been notable in this regard with the latter occurring in patients 10 to 15 years younger in Uganda as compared to the US [15]. The average age of patients with a diagnosis of malignancy in our study was 47.3 years, which is well below those in other countries. All patients who developed DVT in our study had a malignancy with two of the patients being below 30 years old. It would thus appear that malignancy is an important risk factor and is independent of other risk factors such as age and obesity.

BMI was probably the single most important distinguishing factor between our population and the western societies with respect to DVT. Most of our patients were either underweight or of normal weight, and therefore the chances that one would have developed DVT on account of a high BMI are definitely much lower. Obesity has been associated with decreased levels of antithrombin III and fibrinolytic activity with a resultant increase in DVT risk. Surgery in the obese is usually more challenging and will, on average, last longer. In one study, the odds of a postoperative complication increased 12-fold in the obese [16].

History of previously known risk factors was lacking in a large percentage of our patients. For those who had any risk factors, the total number of factors per patient rarely exceeded one. Previous DVT history was particularly difficult to elicit probably because of the low levels of education, lack of past medical records, and poor communication by health workers to patients about their ailments. Other risk factors, such as smoking, have a very low prevalence in Uganda. According to a World Bank data sheet, prevalence of smoking in the UK and US is 10 to 12 times that of Uganda [13]. Other risk factors, such as diabetes mellitus, though increasing at an appreciable rate, are still at relatively lower levels in Uganda compared to western societies. Considering that cancer confers upon a patient undergoing surgery a 3- to 5-fold increase in risk for VTE [17], the relatively low cancer rates may translate into the observed relatively low DVT incidence here.

Diagnosis of DVT was performed using Doppler ultrasound and although many authors agree that ultrasonography may be as good as venography or fibrinogen scan in the diagnosis of proximal DVT, consensus is still lacking concerning the sensitivity of Doppler scan in distal DVT. Many of the studies that have reported high DVT rates among their postoperative patients have employed venography or fibrinogen scanning [12,18] as part of the confirmation of DVT. A significant portion of these high rates of DVT is actually the ‘distal’ DVT, which may be missed by the Doppler scans. Conversely, ultrasound-based studies uniformly report low DVT rates with especially low distal rates.

### Limitations

Our study had several shortcomings. Post-mortems were not performed for the patients who died. Some of the patients were lost to follow-up perhaps leading to an underestimation. D-dimer assays were not included in the study. We were unable to do serial ultrasound evaluations to make the diagnosis of DVT earlier (preoperatively) and determine when DVT is most likely to develop in the postoperative patient. This could have led to an overestimation of DVT due to surgery. Possible recall bias and the lack of past medical records could have made the history obtained from these patients less accurate. Ultrasound scanning findings may be observer dependent; in this study, an experienced sonographer performed the Doppler studies.

## Conclusions

Occurrence of DVT among patients who had major abdominal general surgery in Uganda was low and cancer was the most common associated risk factor.

## Abbreviations

BMI: Body mass index; DVT: Deep venous thrombosis; POD: Postoperative day; VTE: Venous thromboembolism.

## Competing interests

The authors declare that they have no competing interest.

## Authors’ contributions

MG and MAL conceived of the study. MAL collected data. MG wrote the first draft. All authors performed critical reviews for intellectual content and approved the final manuscript.
